# A mixed methods examination of knowledge brokers and their use of theoretical frameworks and evaluative practices

**DOI:** 10.1186/s12961-020-0545-8

**Published:** 2020-03-27

**Authors:** Kristine Newman, Ryan DeForge, Dwayne Van Eerd, Yan Wei Mok, Evelyn Cornelissen

**Affiliations:** 1 Daphne Cockwell School of Nursing, Ryerson University, Toronto, Canada; 2 World Health Innovation Network, Odette School of Business, University of Windsor, Windsor, Canada; 3 Institute for Work & Health, Toronto, Canada; 4 Department of Family Practice, Faculty of Medicine, University of British Columbia, Vancouver, Canada

**Keywords:** Healthcare, Implementation and dissemination, Knowledge broker, Knowledge translation, Mixed methods

## Abstract

**Background:**

Knowledge brokering is a knowledge translation approach that includes making connections between researchers and decision-makers to facilitate the latter’s use of evidence in health promotion and the provision of healthcare. Despite knowledge brokering being well-established in Canada, many knowledge gaps exist, including understanding what theoretical frameworks have been developed and which evaluative practices knowledge brokers (KBs) use.

**Methods:**

This study used a mixed methods design to examine how KBs in Canada (1) use frameworks, models and theories in their practice and (2) how they evaluate knowledge brokering interventions. We gathered interview and survey data from KB practitioners to better understand their perspectives on effective practices. Our analysis focused on understanding the theoretical frameworks used by KBs.

**Results:**

This study demonstrates that KBs in Canada tend not to rely on theories or models that are specific to knowledge brokering. Rather, study participants/respondents draw on (sometimes multiple) theories and models that are fundamental to the broader field of knowledge translation – in particular, the Knowledge to Action model and the Promoting Action Research in Health Sciences framework. In evaluating the impact of their own knowledge brokering practice, participants/respondents use a wide variety of mechanisms. Evaluation was often seen as less important than supporting knowledge users and/or paying clients in accessing and utilising evidence.

**Conclusions:**

Knowledge brokering as a form of knowledge translation continues to expand, but the impact on its targeted knowledge users has yet to be clearly established. The quality of engagement between KBs and their clients might increase – the knowledge brokering can be more impactful – if KBs made efforts to describe, understand and evaluate their activities using theories or models specific to KB.

## Background

Under Canada’s health Act, the health sector is administered and operated on a non-profit basis by the public authority. The public health sector in Canada has been moving toward evidence-informed decision-making for many years [1]. However, ‘knowledge brokering’ roles tend not to be formalised or routine, making research on the effectiveness of KB activities difficult to ascertain. Knowledge brokering is a relatively new approach to knowledge translation in which knowledge brokers (KBs) make connections between researchers and decision-makers to facilitate the latter’s use of evidence in health promotion and the provision of healthcare. KBs focus on information communication, are typically familiar with the field in which they practice and have a high level of credibility in accessing and interpreting research [1–5]. KBs’ responsibilities include finding relevant policy and practice research, synthesising it, organising seminars or meetings, supporting linkages between researchers and decision-makers, and building networks [1–3, 6].

Literature about knowledge brokering in healthcare tends to focus on KBs’ roles. They are seen as linking researchers and knowledge users by developing mutual understandings of goals and cultures [1, 3, 7–9]. Other roles for KBs include knowledge management and building capacity to develop knowledge users’ understanding and skills and to access and apply knowledge [10]. These roles are consistent with Van Eerd et al.’s [11] finding that knowledge brokering involves accessing, adapting and disseminating knowledge as well as linking and networking. Despite existing research on KBs’ roles, there is neither a standard job description nor a list of acceptable qualifications for KBs. One of the reasons for this is the flexible and responsive nature of the role and its contextual influence; moreover, the variety of personal attributes among KBs, combined with the nuanced contextual factors of any given organisation utilising KB assets makes a standardised KB role difficult to define [10].

In a scoping review of the KB literature, Van Eerd et al. found that although KB approaches differ based on stakeholders’ desired outcomes, they do have practices in common [11]. These include disseminating knowledge, linking/networking, adapting/translating knowledge, acquiring knowledge and enhancing capacity. Activities such as assessing and synthesising knowledge were mentioned less often, and evaluation varied from evaluations of KB roles, end-user research use or awareness, to assessments of knowledge-use processes and practice change [11]. Similarly, Bornbaum et al. [10] reported on the variability of knowledge brokering practice and the lack of strong evidence for effectiveness; to date, evidence of impact has been mainly anecdotal or theoretical. The lack of evidence for the impact of knowledge brokering may arise because many KB practitioners do not claim responsibility for their achievements, focusing instead on facilitating knowledge acquisition and/or knowledge utilisation processes with and for their teams [10]. To understand KBs’ effectiveness and to conserve precious healthcare resources, it is important that KBs measure the impact of their interventions and cultivate a culture of evaluation or quality improvement [10, 12].

In developing a taxonomy of implementation theories, models and frameworks, Nilsen [13] suggests that there is an increased proclivity among implementation leads to use models, frameworks and theory to guide their work. Such guidance can help KBs understand and explain how and why particular KB interventions succeed or fail. Models, frameworks and theory can also be used (1) to describe and guide the process of translating research into practice, (2) explain what influences implementation outcomes, and (3) evaluate the implementation [13]. Nilsen reports that, although some scholars are optimistic that using theory will reduce the research–practice gap, others argue that theory is not necessarily better for guidance than common sense [14, 15]. This highlights that the use of theory should not just be an academic exercise but should have real-world applications. However, Davies et al. [16] noted that practitioners were frustrated with the models, frameworks and theories found in the literature. Despite the frustration, they report that some ideas and arguments from the literature did have an impact on practice.

In examining which theoretical frameworks underpinned the studies in their scoping review (research that preceded this current study), Van Eerd et al. [11] found the Linkage and Exchange model [3] to be the most common, while other studies used models such as the Promoting Action on Research Implementation in Health Services (PARIHS) framework [17, 18], the Canadian Health Services Research Foundation model [3], the Knowledge to Action (KTA) model [19], or Rogers theory of Diffusion of Innovations [20]. Almost half of the knowledge brokering approaches examined in their scoping review were guided by multiple frameworks. Similarly, Glegg and Hoens [9] note that the PARIHS framework, KTA model, and Diffusion of Innovations can be used to understand knowledge brokering and its impact.

There are several possible reasons for the use of multiple theories in knowledge brokering research. Kislov et al. [21] found that KBs most often work in interorganisational networks, university–industry collaborations or collaborative research partnerships, and therefore have intermediary and peripheral/boundary positions. Tensions for KBs working in these positions manifest in requirements for different types of knowledge and different types of knowledge brokering. In such situations, where knowledge is transferred to users without being mobilised in routine practice, KBs need to identify and meet clients’ needs to better support evidence-based decision-making at the organisational and policy levels [21]. Similarly, Norton et al. [22] suggest that drawing on multiple theoretical frameworks can inform the function and roles of KBs, what kinds of evidence might be required, and what mechanisms of knowledge synthesis might be deployed.

With respect to the evaluation of KB activities, Maag et al. [23] have argued that having access to practical methodologies to assess the quality of the knowledge brokering is an important concern for some KBs, but that among any such existing frameworks, most tend to be programmatic rather than individual. ‘Developmental evaluation’, for example, aims to balance the critical nature of evaluation with the creative thinking that drives development such that an assessment of impact can combine the rigour of evaluation with the change-oriented and relational roles of organisational coaching [24]. Informed not only by feedback data and by interactions with those a KB supports, but also by an account of organisational changes, developmental evaluation is designed to provide guidance on fine-tuning an implementation and to surface considerations of what conditions hold promise and which ought to be abandoned [24]. In addition to not having a focus on individual knowledge brokering practices, the extent to which KBs have adopted developmental evaluation techniques, however, is not clear in the extant literature – neither the review by Bornbaum et al. [10] nor the one by Van Eerd et al. [11] indicated that functioning and effectiveness are informed or assessed by developmental evaluation techniques.

In sum, there remain significant knowledge gaps relative to how KBs conceive of, inform and assess the impact of their work. Although several scholars have described KBs’ roles and activities [2, 7, 10, 11, 22], to truly understand the impact effectiveness of knowledge brokering and how to sustain it, we need to better understand (1) the extent to which KB practitioners draw on frameworks and theory to inform their practice as well as (2) if and how they evaluate the impact of their activities in terms of meeting knowledge users’ needs and enhancing their capacity to access and use research evidence. These are the aims of the study reported in this paper.

## Methodology

### Study design

The study was approved by Ryerson University’s Research Ethics Board. All data were collected and securely stored in accordance with the Tri-Council Policy Statement and Ethics Board requirements.

The study followed the Convergence Model of mixed-methods design, which features two sets of data – one qualitative and one quantitative – that can be integrated (converged) during analysis to validate, confirm or disconfirm findings [25]. First, we interviewed knowledge brokering practitioners about KB practices and analysed the interview data. Then, we developed a survey questionnaire exploring the same topics as the qualitative phase (e.g. use of frameworks or models; evaluative practices). Key to the design of the study was to ensure that both the qualitative and quantitative methods addressed the same concepts; hence, the generation of survey response items was informed by the findings from the qualitative data. Accordingly, so that survey participants would not be responding to items that were generated from the interview phase, the two data collection procedures were conducted sequentially and the questionnaire was administered to a separate, mutually exclusive sample – as indicated in the Letter of Information, no one who participated in an interview was asked to complete the survey. We compared the two resulting datasets to examine how KBs draw on frameworks and theory and how they approach evaluative practices. Having two datasets enabled us to examine the data by confirming similar and dissimilar findings.

### Study setting, recruitment of participants and sampling

As this study focused on understanding the practices of Canadian KBs, its recruitment strategy focused on KBs practicing in the Canadian healthcare landscape. Selected based on an inclusive list of job titles, contact information of potentially eligible participants was collected from public websites. Potential participants were also sought through knowledge brokering networks and organisations, including the Seniors Health Knowledge Network, Gestalt Collective, Alzheimer’s Society, Physical Therapy Knowledge Broker, British Columbia Rehabilitation Sciences Research Network, Canadian Institutes for Health Research, Michael Smith Foundation, Nova Scotia Health Research Foundation, Social Sciences and Humanities Research Council, Canadian Association for Health Services and Policy Research, Canadian Foundation for Healthcare Improvement, and Strategy for Patient-Oriented Research Support units across Canada.

Once the list of potential interviewees was developed, invitations were emailed to eligible participants. A second invitation was emailed to all who did not respond to the first. Constrained somewhat by limited resources, we aimed to recruit six to eight interview participants who represented a variety of KB practitioners, that is, experienced and new, academic and applied, and from different health systems. Similarly, in the quantitative phase, potential survey respondents were recruited by email using snowball sampling, in which participants in the quantitative phase were asked to recommend professional colleagues. We also recruited respondents through the aforementioned list of Canadian health service organisations and associations that develop and/or employ KBs. The survey was initially distributed to 20 organisations and 23 key knowledge brokering stakeholders. Each email to an organisation or stakeholder included a Letter of Information and, for the quantitative phase, a link to the online survey. Organisations and stakeholders were asked to distribute the email invitation to KBs. Data were collected between November 2014 and October 2015. Only participants whose job title potentially identified them as having a professional KB role in the healthcare field, and who were 18 years of age or older and fluent in English were invited to participate in the interviews or survey.

### The interviews

To guide the design of the qualitative phase, we adopted a constructionist, grounded theory approach [26]. Underpinning this approach is a philosophy that multiple realities exist with respect to social phenomena (i.e. in this case, the processes that are inherent to knowledge brokering) and that researchers come to understand these multiple realities through inquiry and interpretation; that is to say, analysis is informed equally by the data collected and the experience and viewpoint of the analysts. In the qualitative phase, the authors identified as trainees so that we could critically compare interviewees’ insights to our own lived experiences with knowledge brokering. These varied from involvement in hospital quality improvement initiatives to nursing education, to organisational design research. Our aim in the qualitative phase was to distil insights that add to our existing knowledge, and that could be mixed with the data from the subsequent quantitative phase; this reflects a belief that mixed methods research offers complementary datasets wherein strengths from the qualitative research (e.g. rich detail of lived experience) are complemented by strengths from the quantitative research (e.g. potential generalisability) such that the research benefits from more insight than either approach could have created on its own.

Given the geographical diversity of the interview participants as well as the project’s limited resources, interview data were collected by telephone. Consent was obtained from each participant prior to any audio recording being initiated. The research assistant (i.e. a graduate student with basic training in qualitative inquiry) asked all interview questions included in the interview guide (Additional file 1: Appendix A). All interviews were conducted by the same interviewer. Designed to address the research objectives, the interview questions solicited interviewees’ perceptions of how knowledge brokering is defined and enacted. Questions were designed to solicit descriptions of what KBs are, what they do, their use of guiding theory/models, what enables or challenges this use, and opinions and experiences on how KBs could evaluate the success and/or impact of knowledge brokering. Interviews were transcribed verbatim. No additions or revisions were made to the interview guide during the course of data collection.

Analysis of the qualitative data was led by a project team member (RD) with extensive experience in conducting qualitative research and was supported by the lead author (KN). Concurrent with data collection, data were analysed using constant comparison, in which the emergent coding scheme is modified to reflect both newly coded data and existing relevant constructs [26]. By the time the last two transcripts were analysed, evidence of thematic saturation was emerging. The interview dataset lent itself to thematic content analysis because the interviewer followed the interview guide closely in each interview, thus enabling us to extract ‘strips’ of data (e.g. all the data related to the question on defining knowledge brokering; all the data related to the question on using theories/models, etc.). NVivo (Version 9) was used to create ‘nodes’ delineating each strip of data. Line-by-line coding of each strip of data then followed, wherein annotations featured key words or phrases describing the selected portion of an interviewee’s response. Exemplar quotes that offered clear, compelling illustrations of each code were also highlighted. Analysis of each transcript was discussed by RD and KN to identify common insights and to resolve any analytic discrepancies. After coding each transcript, the analysts reviewed the codes assigned, collapsed and renamed the codes to reduce overlap and to enhance clarity, then composed a brief memo summarising that participant’s perceptions of knowledge brokering. Finally, these memos were compared across transcripts and integrated into a single set of findings that convey both the similarities and differences among the participants’ views [25].

### The survey

The survey, in English, consisted of an online, anonymous questionnaire. In accordance with Tri-Council Policy Statement and Ryerson University’s Research Ethics Board requirements, consent to participate in the study was obtained in the first survey question. Designed to provide data to supplement the interview data, questionnaire items were based on the results of a previously conducted scoping review [11] that examined reported approaches, stakeholders and contexts (and outcomes) that KBs use (and achieve) to support healthy aging. After we piloted the survey with five KBs and modified the questionnaire based on their feedback, the final version contained 18 items (10 multiple choice/checkbox questions and 8 open-ended questions). Some of the questions were, “In what type of organisation do you work?” and “How long have you been at this organisation as a Knowledge Broker?” – see Additional file 1: Appendix B. Descriptive statistics were calculated using Excel, and content analysis of the short answers to open-ended questions was completed.

### Comparison of datasets and interpreting data

Comparison of the qualitative and quantitative datasets began after initial analysis of the survey and qualitative data. This was done by bringing together the strengths and non-overlapping weaknesses of quantitative methods with those of qualitative methods. Specifically, in comparing the quantitative and qualitative datasets, we sought to distil quantitatively derived insights that could supplement – or what Creswell calls ‘embellish’ – the qualitative findings by way of getting an indication of how different approaches to theory and to evaluative practices are distributed among KBs. Such analysis enabled us to increase the trustworthiness of our qualitatively derived conclusions [25] and is well suited to this study’s purpose (to examine theoretical and evaluative practices of KBs) in that the analytic aim is to understand knowledge brokering practices by obtaining different but complementary data on the topic.

## Results

This section begins with a description of the study’s participants and respondents. Following that, the findings are arranged in two parts, corresponding to the research objectives: examining KBs’ perspectives regarding (1) the use of models and theories to inform knowledge brokering practices, and (2) KBs’ evaluative practices. Each part draws on both qualitative and quantitative data.

### Description of interview participants and survey respondents

Two mutually exclusive groups of KBs were recruited for this study – one for the interviews and one for the survey. The survey and interview parts of the study were carried out sequentially, wherein the interviews and initial qualitative analysis preceded the quantitative data collection and analysis.

#### Interview participants

A total of eight eligible individuals consented to participate in the qualitative phase from the healthcare sectors in the Canadian provinces of Ontario, British Columbia and Nova Scotia. Interviews lasted between 45 and 75 minutes. To preserve the participants’ anonymity in a field where many knowledge brokers know each other, their characteristics are not reported.

#### Survey respondents

A total of 109 respondents consented to participate. Data from completed questionnaires were excluded from the analysis if participants were not Canadian, did not give consent, or if 50% or more of survey items were not answered. Ultimately, after an analysis of missing data, which was inconclusive, data from 56 respondents (47% of those who responded) were retained for analysis. Respondents self-identified as KBs as all 56 respondents provided an answer to how long they had been a KB and 95% indicated that they were informed by a knowledge brokering approach.

As indicated in Table 1, 63% (35 of 56) of the respondents were from Ontario. Most were early in their careers; indeed, 75% (42 of 56 respondents) indicated they had been in their role for 5 years or fewer. Although there was modest representation from community agencies, governments and not-for-profit organisations working in the healthcare sector, most respondents worked in hospital, educational and research settings.

**Table 1:** Participant characteristics

Characteristic	Response	Number of respondents (%)
Area of employment	Hospital	19 (34%)
	Community & non-for-profit	9 (16%)
	Private sector & public sector	4 (7%)
	Education (university & research)	19 (34%)
	Government & policy	5 (9%)
Length of employment	0–2 years	22 (39%)
	3–5 years	20 (36%)
	6–9 years	7 (13%)
	10+ years	7 (13%)
Work title	Senior management/Director/Manager	15 (27%)
	Educator/Professor/Student	5 (9%)
	Healthcare professional	5 (9%)
	Knowledge (Broker/Exchange/Management/Mobilisation/Translation/Facilitator)	20 (36%)
	Research/Policy	11 (20%)
KB interaction demographics^a^	Patients/Family	20 (36%)
	Community-based sector	31 (55%)
	Researchers	42 (75%)
	Healthcare sector	43 (77%)
	Caregivers	19 (34%)
	Policy-makers	42 (75%)
Location	Prairies	10 (18%)
	Western Canada & Yukon	7 (13%)
	Eastern Canada	3 (5%)
	Ontario & Quebec	35 (64%)

^a^Participants had the option of selecting more than one population with whom they engage in knowledge brokering activities

As shown in Table 1, over a third of respondents reported a job title explicitly related to a knowledge brokering role (e.g. knowledge exchange, management, mobilisation or translation). Another large group of respondents were senior managers or had roles within the research and policy realms of the healthcare sector. Although the stakeholders with whom respondents interacted varied, interactions with researchers, policy-makers and healthcare-sector stakeholders were the most common.

### Use of theories and models to guide knowledge brokering

In an effort to investigate knowledge brokering beyond the focus of existing literature on skills and roles, we examined how KBs’ practices draw on and are informed by theories and models of knowledge translation. Our interpretation of the qualitative data analysis highlighted KBs’ varied application of current knowledge translation models and theories of practice. Some participants clearly arrange their work according to a particular framework. Others more fluidly approach each project without a preselected framework or model in mind and instead rely on knowledge users or paying clients for direction on the need for an organising framework or model.

To determine whether a particular model is suitable for a specific context and is effective in meeting knowledge users’ needs, participants indicated that it is important to thoroughly understand the population with which one works and to select a model that meets the specific needs of the audience. To that end, participants consistently described how they were able to integrate the PARIHS framework and the KTA framework into their interactions in a way that provided conceptual clarity to the project or initiative. For instance, in the case of KTA, the intuitive feeling of progress – from knowledge to action – gives KBs and their clients a process by which they can break down their knowledge brokering initiatives:

“*…particularly for explaining to others what we’re talking about, I’ve found the KTA framework to be really quite useful in helping to thinking about what parts are you’re working on, and what we’re trying to look at and measure, and what components impact adoption or not, or uptake into practice, and the sustainability of that*.” (Knowledge Broker C)

Interview participants also referenced the PARIHS framework, noting that it is considered adaptable and supports the inclusion and examination of different sources of evidence. This framework highlights the importance in knowledge brokering of thoroughly understanding context. Participants also spoke of the PARIHS framework’s simplicity and concreteness:

“*I think probably the one that we land on the most with our clients, and when we’re talking about this, is the PARIHS framework, because it’s just so straightforward and simple*.” (Knowledge Broker G)

“*How we came to use PARIHS is that we found that, when talking about concepts like knowledge translation and knowledge brokering, it was the one that most often we could keep people engaged. A lot of our clients, we kind of lose them when we start talking about conceptual things. The PARIHS framework is concrete: we can talk about it in a really concrete and real way*.” (Knowledge Broker D)

Participants who selected the PARIHS framework to guide their practice reported that they chose it because it is considered effective in building capacity to access and apply research knowledge. Other participants described a hybrid of models as most useful for their practice:

“*I think we’ve sort of created a hybrid model of our own. I use process and determinant frameworks to guide my approach with clinicians. Specifically, using the KTA framework to build a plan of for evidence-informed practice, and PARIHS and/or* [the Theoretical Domains Framework] *to build specific strategies to successfully implement evidence*.” (Knowledge Broker A)

This explicit hybridisation may reflect what many KBs implicitly rely on to drive knowledge brokering, that is, well-known and arguably fundamental knowledge translation frameworks. Moreover, as indicated by the following quote, participants emphasise doing things rather than framing their work with academic models:

“*There’s no magic bullet out there. What is helpful are these multifaceted and likely synergistic efforts. So…you can go down this list and tick off: okay, we’ve got an increasingly supportive client; we have prioritisation methods; we have one-stop shops with evidence services; we have the research evidence tool; we have stakeholder dialogues going on. When all of these things are going on, I think there’s a much greater chance of impacts*.” (Knowledge Broker F)

**Fig. 1:**
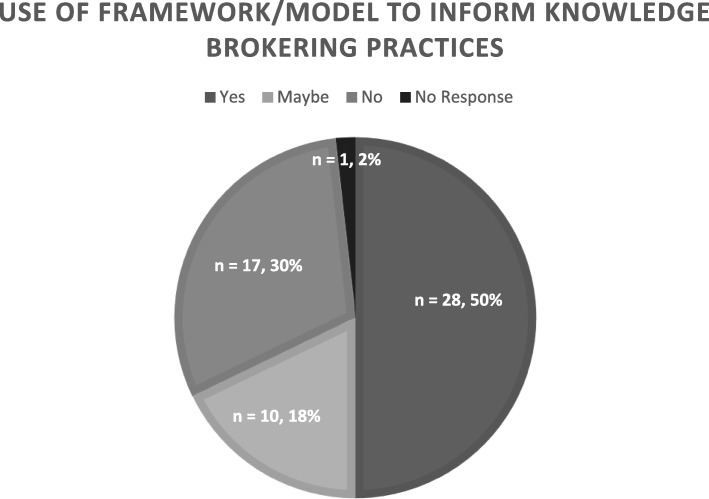
Survey respondents’ use of a framework/model to inform knowledge brokering practices

Findings from the survey support the claim that knowledge brokering practices tend to be informed by ‘an approach’, and model selection varies depending on context and situation (Fig. 1). As with interview participants, survey respondents reported using the KTA and PARIHS frameworks most frequently.

Comparison of the qualitative and quantitative findings shows that not only are KBs in this study familiar with the fundamentals of knowledge translation theory, they either implicitly or explicitly draw on process models (e.g. KTA) or determinant frameworks (e.g. PARIHS) to guide their knowledge brokering. Moreover, these findings seem to indicate that KBs in the study do not rely on only one model or framework; rather, they consider the contexts in which their KB work takes place and draw on models and frameworks that suit those contexts as well as their knowledge users’ needs.

### Evaluative practices

Interview participants reported a range of evaluative practices, ranging from none at all to passive evaluations, to ‘wait and see’ approaches, wherein changes to policy and/or practice were taken as indicators of supposedly successful knowledge brokering. While not necessarily representative of typical knowledge brokering ‘success’, the following quotes effectively illustrate that in some cases, changes in government policy are taken as indications of effective knowledge brokering:

“*Well, a lot of the stuff that we do, it’s fairly immediate and visible. When it goes up to Cabinet, you can then see the release; the nature of the policy that is put forward comes out of it*.” (Knowledge Broker F)

“*We’ve seen a number of small policy changes happen in the African context. The consequence of policy dialogues and evidence briefs, malaria policy change – we’ve seen liberal health policy changes*.” (Knowledge Broker A)

Indeed, it may take quite some time for the benefits of knowledge brokering to manifest, if at all:

“*What we will know in a couple of years is, you know, will we see the rates going down? We are able to evaluate – and we have those qualitative measures in place, and* [if] *there hasn’t been a big uptake of what we have been recommending, then we will be able to assess that that didn’t work*.” (Knowledge Broker E)

Such findings can be interpreted as a means by which KBs operationalise the impact of their work. With more of a focus on the evaluation of how knowledge brokering services were provided, other KBs who regard their practices as what they call “*customer service units*” take client satisfaction as the best indicator of success:

“*When something hasn’t gone right, we hear about it. We are a customer service unit, so we hear about it if our service isn’t appreciated. So that* [feedback] *is coming from our faculty members who are involved in our service, as well as our community partners who are involved in our service*.” (Knowledge Broker C)

“*Well it’s easy for us, because the only way we’re keeping score is that we keep getting clients. We have a fruitful business, and have* [been] *for four or five years now. And yeah, I’m kind of joking, but I do think that* [repeat business] *is an indicator of successful approaches*.” (Knowledge Broker H)

Others take a return to the status quo as an indication that knowledge brokering had little impact:

“*…when there’s no lasting change. So a lot of the times, this work is a research study. And you get people on board and they participate. And you do this, and you do that, um – nothing really formal gets put into place. Work happens, but nothing really formal happens to really change culture and context. So, when the study’s over, it all just goes back to the way it was before*.” (Knowledge Broker D)

**Fig. 2:**
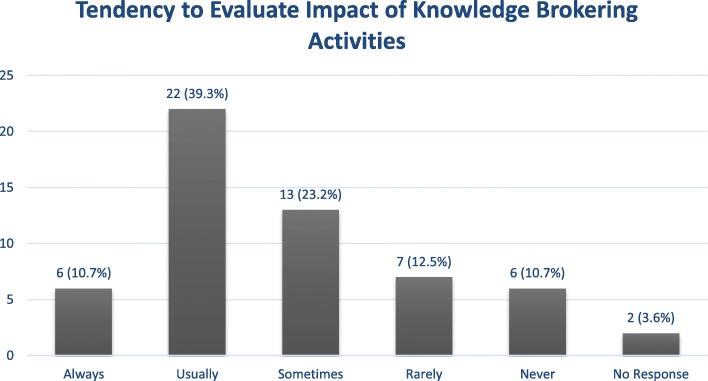
Survey respondents’ tendency to evaluate impact of knowledge brokering

These qualitative data suggest that KBs’ evaluative practices vary significantly, from tracking health outcome and policy changes, to those that are somewhat informal and unstructured. The survey data confirmed this finding. As shown in Fig. 2, 23% of respondents indicated that they ‘Never’ or ‘Rarely’ evaluate the impact of their knowledge brokering practices; a similar portion (23%) ‘Sometimes’ do, whereas only 50% responded that they ‘Usually’ or ‘Always’ evaluate their practices.

**Fig. 3:**
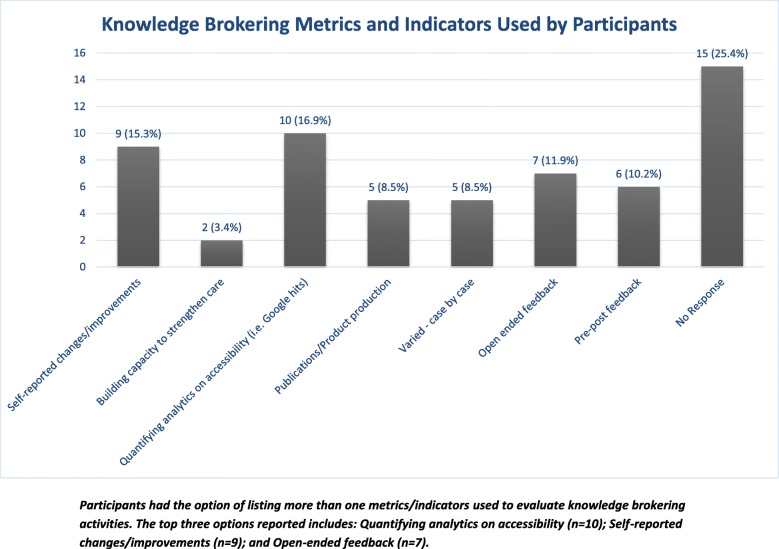
Knowledge brokering metrics and indicators of success

For respondents who do assess the impact of their knowledge brokering practices, the specific metrics or indicators they use are shown in Fig. 3. Notably, the most common indicators of success are producing knowledge products that are used, as measured by, for example, the number of website visits and file downloads.

When asked in the survey to describe what outcomes are most important for assessing knowledge brokering success, most respondents chose research evidence being used in decision-making. Less frequently chosen indicators of success were newly created partnerships characterised by two-way knowledge exchange, and evidence of changes in policy or practice. Although respondents’ recognition that outcomes are important in knowledge brokering is perhaps not surprising, there appears to be a lack of formal, structured knowledge brokering evaluation. More common are informal assessments that assume that if partnerships between knowledge users and KBs were created, research evidence will be used in decision-making; ultimately, the partners (i.e. KBs and knowledge users) wait to see if changes in practice or policy emerge.

## Discussion

The findings from our mixed-method study show that the Canadian samples of self-identified KBs describe their roles and skills in ways consistent with existing literature [2, 7, 10, 11, 22]; that is, as our qualitative data have shown, KBs build relationships that enable knowledge users to gain access to research evidence that can inform or improve their practices. Against this backdrop, both our qualitative and quantitative data suggest, at least among the KBs in our study, that the use of theories and frameworks to guide knowledge brokering varies. In addition, our findings show that the evaluative practices of these KBs also vary (and, for some, are negligible). Those who do evaluate their knowledge brokering do so informally, often by (1) seeking to identify demonstrable changes in policy and/or practice (assuming them to be attributable to the knowledge brokering intervention) and/or (2) sustaining revenue from the clients who use their knowledge brokering services.

In contrast to these informal evaluative practices, formal evaluations should aim to, first, assess knowledge brokering impact in terms of practice and/or policy change and, second, determine how and to what extent particular knowledge brokering activities helped achieve those outcomes. The literature on evaluative practices, especially programme evaluation, has proliferated in the past decade [27–29], and includes widely accepted guidance on developing project-appropriate logic models, outcomes and outcome indicators. Dobbins et al. [30] recently found that a knowledge translation intervention delivered by KBs resulted in improvements in evidence-informed decision-making knowledge, skills and behaviours, suggesting that, if KB researchers develop concrete, actionable indicators and ways to measure them – informed by theories, models or frameworks and keeping in mind a wide range of stakeholder perspectives – perhaps a culture of evaluation can grow within knowledge brokering. A culture of evaluation may further define knowledge brokering roles and qualifications, which may be of use to organisations as they create knowledge brokering positions and related job descriptions.

This study’s findings about the use of theory or models to guide knowledge brokering are consistent with what Van Eerd et al. [11] identified in their scoping review – there appears to be much variability, which may reflect KBs’ efforts to find theories or models that match knowledge users’ context and needs. Both our qualitative and quantitative findings point to two frequently endorsed frameworks: the KTA model [19], which Nilsen et al. categorise as a process model [13], and the PARIHS framework [17, 18], seen by Nilsen et al. as a determinant framework. KBs’ familiarity with KTA and PARIHS may reflect their familiarity with the fundamentals of knowledge translation. As a process model, KTA is deemed useful in describing to clients the steps of acquiring, tailoring and applying research evidence [19]. Similarly, PARIHS, with its emphasis on understanding how successful implementation is determined by facilitation strategies, robustness of evidence and by the context in which change is being introduced, provides KBs with three concrete, intuitive realms in which to focus brokering [17, 18]. The absence in our findings of models or theories specific to knowledge brokering can perhaps be understood as a reflection of knowledge brokering being one knowledge translation strategy. It appears that, among our study’s samples, KBs’ aim in knowledge brokering is to reach knowledge translation goals; hence, they may rely on knowledge translation frameworks (such as KTA or PARIHS) rather than knowledge brokering theories.

Nilsen et al.’s [13] three approaches to the use of theory among knowledge practitioners might be useful to KBs. These are describing the process of translating research into practice, understanding or explaining what influences implementation, and evaluating it. Were KBs able to clearly identify into which of the three approaches their clients’ needs fit by describing, understanding or evaluating implementation, then perhaps KBs could more readily and accurately hone in on the model, theory or framework that best matches their knowledge users’ evidence needs. If the end users that KBs are serving do not explicitly ask for an evaluation of impact, it may be that the extent of KBs' theorization is limited to describing and understanding the intervention and the evaluative aspects of popular frameworks like the KTA and PARISH are under utilized. Inconsistent and, in some cases, non-existent evaluative practices in knowledge brokering remain a concern, especially in light of others’ assertions that knowledge brokering has not robustly demonstrated its effectiveness as a knowledge translation strategy that builds capacity to access and apply research knowledge [10].

Our study has several limitations as well as strengths. Given the dearth of literature about KBs’ use of theories, models and frameworks, we had hoped that the qualitative phase of our study would yield data-rich insights into how KBs use theory. Instead, our findings describe which theories are used, and in which circumstances. Future research could delve further into KBs’ use of theory, especially with respect to which KB-practice decisions are informed by it, and how explicitly used theories interact with the theories implicit in KBs’ tacit knowledge bases, and how one’s theoretical orientation impacts the way(s) in which knowledge brokering work is evaluated. Another limitation of our study is the small sample of interview and survey participants. Although we observed theoretical saturation in the eight interview transcripts, a larger sample may have yielded more diverse views. Conversely, our achievement of theoretical saturation suggests that the qualitative phase addressed core concepts in answering our research questions. Indeed, our focus on KBs’ use of theory and their evaluative practices may have contributed to this saturation. We also cannot rule out selection bias given the small sample size and recruitment methods. Other strengths of the study include its mixed-method design, which compared qualitative and quantitative findings on the same topics to create a more comprehensive, in-depth understanding of the phenomenon being examined and to enhance the findings’ credibility. Furthermore, we believe, as authors and analysts, that by giving credence to our own lived experiences with knowledge brokering, we were able to further enhance the credibility of the findings; for example, our own familiarity with the PARIHS and KTA frameworks facilitated a deeper understanding and appreciation of our participants’ responses than could have been achieved by a researcher from outside of the field. Similarly, our experiences with projects that have been robustly evaluated as well as with projects that lack a strong evaluation resonated strongly with the data provided by the participants; our critical analysis and discussion of the findings was strengthened by such resonance.

## Conclusions

Our study has several implications for knowledge brokers that build on Nilsen et al.’s [13] assertion that it is useful to take a three-pronged approach to the use of theory in knowledge brokering. First, we encourage KBs to integrate evaluative practices into their work, so that the outcomes that result from their engagement are assessed using appropriate, accessible outcome measures. Second, to understand whether and how knowledge brokering is effective, the evaluation plans of knowledge brokering projects might include objectives, activities, outputs and outcomes specific to knowledge brokering.

As a form of knowledge translation, knowledge brokering continues to expand, but it has yet to clearly establish what impact it has on its targeted knowledge users with respect to their capacity to access and apply research evidence. Engagement between KBs and their clients might improve if KBs made efforts to engage their knowledge users in discussions that explicitly describe the processes being considered to translate research into practice, which may in turn help KBs and knowledge users alike better understand what factors influence the implementation of these processes, and how to best develop an appropriate, robust evaluation plan. Such knowledge brokering practices would reflect an understanding that the needs of knowledge users and clients are not exclusive of the need to incorporate theoretical and evaluative activities; balancing practical and theoretical/evaluative imperatives is a goal to which knowledge brokering practitioners might aspire.

## Supplementary information

**Additional file 1: Appendix A.** semi-structured interview guide. **Appendix B.** Online survey questions.

## Data Availability

The datasets analysed during the study are available from the corresponding author on reasonable request.

## Abbreviations

KBs: Knowledge brokers
KTA: Knowledge to Action model
PARIHS: Promoting Action on Research Implementation in Health Services framework
