# Supplementary material for: A mixed methods examination of knowledge brokers and their use of theoretical frameworks and evaluative practices
Source: Health Res Policy Syst. 2020 Mar 27;18:34. doi: 10.1186/s12961-020-0545-8 (PMC7099818; doi:10.1186/s12961-020-0545-8)
Supplement: Supplementary file 1 — Additional file 1: Appendix A. semi-structured interview guide. Appendix B. Online survey questions. [file 12961_2020_545_MOESM1_ESM.docx]

**Appendix A: Interview Guide**

**Topic One: Orienting to KB**

1. To what extent do you consider yourself a knowledge broker?
2. How would you define knowledge brokering?
3. What skills does a knowledge broker require?
4. What resources do knowledge brokers use?
5. What is the role of a Knowledge Broker?

**Topic Two: Your own KB practice**

1. How do you conduct your Knowledge Brokering role?
2. What theories or models guide your practice?
3. Is there a particular theory or model that guides your practice? If so, what is it?
4. How did you come to select that particular model?
5. Who are your knowledge end-users (i.e. those people requesting information)?
6. Can you give me a for instance of a knowledge brokering activity you did recently?
7. Can you give me another for instance of an activity that is of a different nature?
8. How did you and your knowledge users arrive at a common understanding of what KB could/does entail?
9. How do you know if/when your knowledge brokering practice has been ‘successful’?
10. What might indicate to you that it hasn’t worked as well as you’d hoped?
11. What conditions need to be in place in order for knowledge brokering to work within and among a group of health care practitioners?
12. What conditions might preclude ‘successful’ knowledge brokering?

**Topic Three: KB and Healthy Aging**

1. In what circumstances does knowledge brokering support the context of healthy aging?
2. Do you see a role for knowledge brokers for the aging population? If so, describe.
3. To what extent does your knowledge brokering practice contribute to the healthy aging of Canadians?
4. What would you say are the key ingredients to successful knowledge brokering?
5. How are those ingredients salient to the context of healthy aging?

Probes:

1. What are your experiences with being introduced to and/or selecting and using different models of knowledge brokering?
2. What are your reflections on knowing if a particular model is suitable for its context and effective in meeting knowledge users’ needs?
3. What models work within the context of supporting healthy aging?
4. What is the most effective way to meet the knowledge needs of care providers and the aging population?

**Appendix B: Online Survey Questions**

1a) What is your work title?

______________________

1b) In what type of organization do you work? (Choose one)

- Hospital
- Community
- Policy
- Private Sector
- Other __________________________

1c) How long have you been at this organization as a Knowledge Broker? (Choose one range)

- 0-2 years
- 3-5 years
- 6-9 years
- 10+ years

1d) Who do you interact with or assist when Knowledge Brokering? (Choose all that apply

- Patients/family
- Community based sector
- Researchers
- Health care sector
- Caregivers
- Policy makers

1e) Do you work in an area that impacts the aging population?

- Yes
- No
- Maybe

1f) If yes, how do you impact the aging population?

______________________

2a) Is your position informed by a Knowledge Brokering Approach? (Examples: Knowledge Exchange, Behavioural, Linkage and Exchange, Research/Evidence Synthesis, etc.)

- Yes
- No
- Maybe

2b) If yes, what kind of Knowledge Brokering approach? (Choose all that apply)

- Knowledge Exchange
- Behavioural
- Linkage Exchange
- Research/Evidence Synthesis
- Other __________________________

2c) Do you use any sort of framework or model during Knowledge Brokering activities?

- Yes
- No
- Maybe

2d) If yes, how do you use models in your Knowledge Brokering activities?

______________________

3a) What are the top three Knowledge Broker activities you perform on a regular basis?

______________________

3b) Of the Knowledge Broker activities you perform, which do you think generally have the most impact?

______________________

4a) Do you ever evaluate the impact of your Knowledge Brokering? (Choose one)

- Always
- Usually
- Sometimes
- Rarely
- Never

4b) If you ever evaluate, what specific metrics or indicators have you used?

______________________

4c) If you ever evaluate, how were these metrics or indicators measured?

______________________

4d) What specific metrics or indicators do you believe are useful toward measuring the impact of Knowledge Brokering? (Choose all that apply)

- Number and nature of requests for information
- Number and nature of requests for linkage
- Participation at meetings
- Number of and level of representation at workshops and other Knowledge Translation events with stakeholders
- Number of presentation team members (including researchers, clinicians, decision-makers, policy-makers) makes to their own organizations/institutions
- Number and interdisciplinary nature of new study proposals developed
- Number and interdisciplinary nature of presentations at research conferences and publications in peer-reviewed journals
- Number and nature of new provincial, national and international partnerships/collaborations
- Researcher, decision-making, and other stakeholders' views on success
- All of the above
- Other __________________________

4e) What outcomes do you think are important for successful Knowledge Brokering? (Choose all that apply)

- Creation of partnerships with evidence of two-way exchange processes (e.g., where researchers learn from decision-makers/stakeholders and vice-versa)
- Progression of "new emerging team" into an effective and collaborative research team responsive to stakeholder needs/priorities
- Use of research evidence in decision-making (e.g., policy, health services, delivery, clinical practice)
- Changes in practices or policies
- All of the above
- Other __________________________

5) Finally, based on your experience, what do you believe are the key facilitators to successful Knowledge Brokering?

______________________
